# Oral dysaesthesia: a special focus on aetiopathogenesis, clinical diagnostics and treatment modalities

**DOI:** 10.1038/s41415-024-7057-9

**Published:** 2024-02-23

**Authors:** Arwa M. Farag, Barbara Carey, Rui Albuquerque

**Affiliations:** 4141526222001 Associate Professor, Department of Oral Diagnostic Sciences, Faculty of Dentistry, King AbdulAziz University, Jeddah, Saudi Arabia; Division of Oral Medicine, Department of Diagnostic Sciences, Tufts School of Dental Medicine, 1 Kneeland St., 6th floor, Boston, MA 02111, USA; 4141526222002 Consultant in Oral Medicine, Department of Head and Neck Surgical Oncology, Guy´s and St Thomas´ Hospital NHS Foundation Trust, London, UK; 4141526222003 Consultant in Oral Medicine/Honorary Clinical Senior Lecturer, Guy´s and St Thomas´ Hospital NHS Foundation Trust, King´s College London, Oral Medicine Department, Floor 22, Tower Wing, Guy´s Hospital, Great Maze Pond, London, SE1 9RT, UK

## Abstract

Oral dysaesthesia is a condition characterised by persistent alteration to oral sensation, perceived by the patient to be abnormal and/or unpleasant, in the absence of any mucosal pathology. The condition can be difficult to detect and diagnose. A possible peripheral or central neuropathic aetiology has been proposed. Burning mouth syndrome (BMS) is the most common idiopathic oral dysesthesia in which long-term suffering is often reported by patients. Recent efforts from professional organisations and study groups have provided a consensus on BMS disease definition and diagnostic criteria. Large-scale epidemiological studies are required to provide an accurate estimate for prevalence and incidence of the condition. Meticulous diagnostic investigations which may require interdisciplinary teamwork are often warranted to reach an accurate diagnosis. A combination of interventional modalities, with a holistic approach, is key for successful management and improvement in patients' quality of life.

## Key points

- Oral dysaesthesia is defined as an idiopathic chronic condition, characterised by persistent alteration to oral sensation, perceived by the patient to be abnormal and/or unpleasant, in the absence of an identifiable local or systemic cause.
- Burning mouth syndrome is the most commonly encountered idiopathic oral dysesthesia in a dental setting. Thorough knowledge of burning mouth syndrome diagnostic criteria and the correct implementation of diagnostic investigations are essential for establishing the correct diagnosis.
- Holistic and multi-modal management strategies are key for successful management of oral dysaesthesia.

## Introduction

Oral dysaesthesia (OD) is defined as an idiopathic chronic condition, characterised by persistent alteration to oral sensation, perceived by the patient to be abnormal and/or unpleasant, in the absence of an identifiable local or systemic cause.^1^^,^^2^^,^^3^ A variety of terms have embraced OD, including but not limited to: stomatopyrosis, glossopyrosis, stomatodynia, glossodynia, sore mouth, sore tongue and burning mouth syndrome (BMS).^3^ In their definition of BMS in 2018, the World Health Organisation embraced the concept of a dysaesthesia sensation.^4^This was also observed by the Orofacial Pain Classification Committee when defining BMS as ‘an intraoral burning or dysaesthesia sensation, recurring daily for more than two hours per day for more than three months, without evident causative lesions on clinical examination and investigation'.^5^ Considered a neuropathic pain, the evidence remains low to support any specific treatment in preference to another and consequently, decision-making is predominantly based on clinical expertise.^6^^,^^7^ Clinicians need to undertake multiple investigations to establish the diagnosis, as well as consider overall management options, before referring to a specialist centre. The aim of this paper is to provide an up-to-date guidance on the diagnostic modalities and management approaches for OD (with a focus on BMS), providing a foundation for general medical and dental practitioners alike.

## Clinical characteristics and aetiology

Although symptoms are classically described as ‘pains', it is pivotal to note that many describe BMS as spontaneous tactile and/or thermal sensations, such as numbness, tingling, pricking, burning, or a shooting sensation.^8^^,^^9^ BMS predominately affects the anterior one-third of the tongue and hard palate, but many patients also describe involvement of the lips and labial mucosa.^10^^,^^11^^,^^12^ The aetiology of BMS is yet to be determined^13^ but a combination of neurological and psychological factors have been proposed.^13^^,^^14^ The psychiatric literature has reported a reasonable body of evidence documenting psychosomatic comorbidities in patients with oral dysesthesia, including insomnia, hypochondriasis and gastrointestinal disturbances.^10^^,^^11^^,^^12^ Cognitive-behavioural and affective-motivational factors, such as depression, anxiety, fear, and past experiences, may further complicate nociceptive interactions and sensory perception.^11^^,^^12^

## Epidemiology

There does not appear to be any literature concerning epidemiology of OD collectively.^13^ Many studies focus on BMS, though the precise estimate on prevalence and incidence is difficult to establish given the historic lack of consensus on disease definitions and diagnostic criteria.^15^ The epidemiology statistics provided in the literature before the release of the *International classification of orofacial pain* 2020 should be interpreted with scepticism. Overall, OD symptoms are rare and occur in less than 1% of the population.^13^ Restricting symptoms to BMS only, the range varies between 0.7-4.6% of the adult population.^16^^,^^17^^,^^18^^,^^19^ There is a higher predominance in women and specifically during peri-/post-menopausal age. Several studies show psychosocial comorbidities similar to those of other persistent pain conditions.^15^^,^^20^

## Diagnostics

Ordinarily, the diagnosis of OD is a diagnosis of exclusion, and eliminating the possibility of a pre-existing underlying cause with the following investigations remains the gold standard.^15^

### History and clinical examination

A focused history combined with detailed chairside examination are the most pivotal tools to formulate a list of differential diagnoses and subsequent investigations, which will lead to an accurate diagnosis.^21^ Establishing the symptoms using ‘SOCRATES' (site, onset, character, radiation, associations, time course, exacerbating/relieving factors, severity) provides a launchpad for focusing the clinical examination.^21^

The extra-oral examination should include inspection and palpation of the head and neck. Intra-orally, the mucosa, periodontium and dentition must be assessed to exclude mucosal lesions, odontogenic pathology and/or parafunctional habits. Referral to ear, nose and throat specialists may be required if objective evidence of altered or reduced taste is noted.^22^ General dental practitioners (GDPs) should explore for objective evidence of a cranial nerve deficit, including anaesthesia or paraesthesia (with or without extra-oral features), as this may warrant neurology referral. It is essential they can distinguish a routine case of OD from a more unusual presentation that requires urgent care referral. The *British Medical Journal* provides a comprehensive pathway of investigations and overall management of oral dysgeusia, with further guidance of when to refer urgently.^19^ Other symptomatology that requires referral to secondary care includes unilateral burning sensation or altered sensation, burning involving the posterior tongue, dysphagia, odynophagia and weight loss.^19^^,^^22^

### Clinical and laboratory investigations

Depending on clinical findings, further clinical investigations may be warranted and are commonly undertaken in secondary care. Investigating the current list of the patient's medications might uncover the offending medication causing the oral dysesthesia (that is, angiotensin-converting enzyme inhibitors, 5-hydroxytryptamine reuptake inhibitors, hormone replacement therapies). Laboratory testing is an essential tool to reveal underlying blood dyscrasias/nutritional deficiencies (for example, deficiency of iron, folic acid, and/or vitamin B12), autoimmune disorders causing thinning of the oral mucosa or ulceration and hormonal imbalances (ie hypothyroidism, cortisol imbalance, or oestrogen imbalance).^15^ Viral culture and fungal culture/cytology may be appropriate when suspecting oral infections.^15^ Biopsy remains the gold standard in diagnosing a plethora of oral conditions and can be further aided by special stains and immunohistochemistry. Sialometric analysis, including stimulated and unstimulated salivary flow, can objectively diagnose those suffering from oral burning due to hyposalivation. Table 1 depicts the clinical and laboratory workup that may uncover a pre-existing systemic condition resulting in OD. A Delphi study by Charlotte *et al.* looked for a consensus on what tests should be undertaken in BMS patients in randomised control trials.^23^ As OD embraces a wider number of symptoms, from burning to altered taste to dryness, no official guidance is available for diagnostic testing. The clinician will need to tailor investigations on a case-by-case basis.

**Table 1:** Clinical, imaging and laboratory investigations to uncover existing systemic condition causing oral dysesthesia

	Differential diagnoses to be ruled out	Examples	Diagnostic modality
BMS	Hyposalivation	Drug-induced hyposalivation Sjögren's syndrome	Stimulated and unstimulated sialometry Sjogren's syndrome ACR-EULAR diagnostic criteria^42^ American College of Rheumatology (ACR) and the European League Against Rheumatism (EULAR)
	Oral fungal infection	Oral candidiasis	Cytology smear or biopsy
	Oral viral infections	Primary and recurrent herpes simplex or varicella zoster infections	Clinical presentation, culture and sensitivity
	Autoimmune or immune derived oral mucosal lesions	Aphthous ulcers and oral lichen planus Mucous membrane pemphigoid and pemphigus vulgaris Lupus erythematosus	Empiric therapy Biopsy with immunohistochemistry Antinuclear antibody, anti-double-stranded DNA, anti-Smith antibody
	Anemias	Iron, vitamin B12 and folic acid deficiencies	Complete full blood count (FBC) with haematinics (iron, ferritin, vitamin B12 and folic acid levels)
	Metabolic disorders	Diabetes mellitus	HbA1c
	Nutritional deficiency	Zinc deficiency	Serum zinc level
	Endocrine disorders	Hypothyroidism Cortisol imbalance Oestrogen imbalance	Serum thyroid-stimulating hormone, triiodothyronine, thyroxine Serum cortisol Serum 17-beta oestradiol levels
	Gastrointestinal disorders	Crohn's and ulcerative colitis	Endoscopy/colonoscopy Routine blood tests (FBC, haematinic) Inflammatory markers (erythrocyte sedimentation rate, C-reactive protein) Serum anti-neutrophil cytoplasmic antibodies and anti-saccharomyces cerevisiae antibodies
		Gastro-esophageal reflux	Empiric therapy or endoscopy
	Allergies and hypersensitivity reaction	Oral contact allergic stomatitis/lichenoid reaction	Elimination of the suspicious agent and/or patch test
		Drug-induce lichenoid reactions	Cessation of the offending medication and/or biopsy

### Imaging investigations

Imaging via computed tomography (CT) or magnetic resonance (MRI) may have a role in OD where there is objective evidence of cranial nerve deficits. In cases with objective altered taste on a background of chronic rhinosinusitis, CT of the nasal cavity and paranasal sinuses may be warranted.^24^

Expanded imaging of the head to include the brain may uncover life-threating pathologies such as central nervous system tumours, aneurysms, arteriovenous malformation and multiple sclerosis.^25^^,^^26^ MRI to explore neurological changes in patients with altered taste has been advocated.^27^ MRI in those with BMS has been widely explored. Khan *et al.* showed changes in the structure and function in the medial prefrontal cortex and hippocampus in MRI studies of BMS patients, implicated in regulating mood and depressive symptoms.^28^ Adamo *et al.* reported higher frequency of white matter hyperintensities in patients with BMS.^29^ Imaging should be prioritised if the pain manifests in a non-classical manner or if accompanied by other unexplained complexities (that is, sensory/motor deficits or autonomic phenomena, unilateral symptoms etc). Table 1 details the diagnostic imaging modalities that may reveal an underlying pathology implicated in OD symptoms.^26^

### Adjunct investigations

Quantitative sensory testing has been useful when addressing pain perception in patients with BMS and other neuropathic pains.^30^ Pain/mechanical/thermal detection thresholds, as well as dynamic mechanical allodynia, manifest significant abnormalities in the formerly mentioned conditions.^31^^,^^32^^,^^33^ Electrogustometry testing using taste/tingling detection thresholds ratio may also be helpful in the diagnosis of BMS.^33^However, the low specificity renders these modalities less reliable for diagnosing neuropathic orofacial pain.^34^ From a clinical perspective, these instrument-based diagnostics are better suited for research rather than clinical practice.

### Psychological and sleep considerations

The bidirectional association between psychological/sleep comorbidities and OD/BMS is well established.^35^^,^^36^^,^^37^ It is difficult to differentiate if the psychological comorbidity is the causative/perpetuating factor for the OD symptoms or is a consequence of long-term suffering due to the oral condition. Therefore, it is recommended to consider these influential factors when attempting to diagnose OD. A number of well-validated psychometric inventories exist, including the Symptom Checklist-90-Revised (SCL-90R), Hospital Anxiety and Depression Scale (HADS) and Hamilton Anxiety and Depression Scale (HAM-A and HAM-D).^38^ GDPs are in a strategic position to identify suicidal risk or severe depression or anxiety. Secondary care assessment is essential in these cases.^38^ Furthermore, sleep quality can be evaluated using the Pittsburgh Sleep Quality Index, Epworth Sleepiness Scale (ESS) and Medical Outcomes Study Sleep Scale.^39^ These scales help the clinician make an informed decision regarding the need to refer their patients for comprehensive psychiatric and/or sleep evaluation.

## Management

### Therapeutic modalities in the management of oral dysaesthesia

Topical interventions, psychotropic medications and psychological therapy remain at the forefront in the armamentarium for the management of OD.

Multiple systematic reviews addressing topical and systemic interventions in BMS have been published but no single recommended treatment has been proposed.^6^^,^^40^ A review of the various therapeutics, dosages and risks associated with topical and systemic medications is available in previous work published by the current authors.^6^^,^^40^ The evidence favouring one treatment over another is currently limited, with clinician experience and patient values playing significant roles. ^6^^,^^40^ Table 2 covers a wide range of interventions, with a particular focus on topical and systemic agents, that have shown efficacy in managing BMS. These interventions can also be trialed in those presenting with other OD symptoms.

**Table 2:** Therapeutic modalities

Category of intervention	Therapeutic modalities
Topical interventions	Clonazepam 0.5 mg/mL solution
	Clonazepam 1 mg disintegrated table
	Capsaicin oral rinse at 0.02%
	Aloe vera gel
	Bupivacaine lozenge
Systemic interventions	Clonazepam 0.5-1 mg
	Tricyclic antidepressants (amitriptyline 10-50 mg; nortryptiline10-50 mg)
	5-hydroxytryptamine-noredrenaline reuptake inhibitors (duloxetine 60 mg, venlafaxine 75-150 mg)
	Anticonvulsants (gabapentin 300-2,400 mg, pregabalin 150-300 mg)
	Alpha-lipoic acid
	Trazodone
	Melatonin
Miscellaneous interventions	Photobiomodulation therapy
	Repetitive transcranial magnetic stimulation of the motor cortex
	Cognitive behavioural therapy

Addressing underlying anxiety, depression and other mental health disorders has resulted in an overall improvement of symptoms.^41^ While the majority of these studies were conducted for those presenting with BMS, they also provide good support for use in other OD cases. Cognitive behavioural therapy - a talk therapy focusing on the principle that mental wellbeing is a result of a connection between a person's thoughts and behaviours^41^ - has also been embraced as part of overall patient management.

## Conclusion

BMS is just one of the conditions under the umbrella of OD. OD remains difficult to detect, diagnose and manage. A lack of awareness within the health care community regarding OD and the scarcity of specialists trained to manage these conditions compounds patient frustration and suffering in their journey with this condition. A holistic approach enables clinicians to appropriately assess the patient and tailor treatment accordingly. Recognising and addressing any functional and/or psychological contributors is fundamental for successful management.
